# Molecular, Cellular, and Technical Aspects of Breast Cancer Cell Lines as a Foundational Tool in Cancer Research

**DOI:** 10.3390/life13122311

**Published:** 2023-12-08

**Authors:** Brittany L. Witt, Trygve O. Tollefsbol

**Affiliations:** 1 Department of Biology, University of Alabama at Birmingham, 902 14th Street, Birmingham, AL 35228, USA; bwitt95@uab.edu; 2 Integrative Center for Aging Research, University of Alabama at Birmingham, 1530 3rd Avenue South, Birmingham, AL 35294, USA; 3 O’Neal Comprehensive Cancer Center, University of Alabama at Birmingham, 1802 6th Avenue South, Birmingham, AL 35294, USA; 4 Nutrition Obesity Research Center, University of Alabama at Birmingham, 1675 University Boulevard, Birmingham, AL 35294, USA; 5 Comprehensive Diabetes Center, University of Alabama at Birmingham, 1825 University Boulevard, Birmingham, AL 35294, USA; 6 University Wide Microbiome Center, University of Alabama at Birmingham, 845 19th Street South, Birmingham, AL 35294, USA

**Keywords:** breast cancer, cell lines, molecular pathways, epigenetics, xenograft, cancer prevention, cancer therapy

## Abstract

Breast cancer comprises about 30% of all new female cancers each year and is the most common malignant cancer in women in the United States. Breast cancer cell lines have been harnessed for many years as a foundation for in vitro analytic studies to understand the use of cancer prevention and therapy. There has yet to be a compilation of works to analyze the pitfalls, novel discoveries, and essential techniques for breast cancer cell line studies in a scientific context. In this article, we review the history of breast cancer cell lines and their origins, as well as analyze the molecular pathways that pharmaceutical drugs apply to breast cancer cell lines in vitro and in vivo. Controversies regarding the origins of certain breast cancer cell lines, the benefits of utilizing Patient-Derived Xenograft (PDX) versus Cell-Derived Xenograft (CDX), and 2D versus 3D cell culturing techniques will be analyzed. Novel outcomes from epigenetic discovery with dietary compound usage are also discussed. This review is intended to create a foundational tool that will aid investigators when choosing a breast cancer cell line to use in multiple expanding areas such as epigenetic discovery, xenograft experimentation, and cancer prevention, among other areas.

## 1. Introduction

Breast cancer cells have been utilized for over 50 years to establish various techniques to give prognosis and treatment for breast cancer. Many well-known mechanisms have been utilized and analyzed with the use of breast cancer cell line research. For example, Trastuzumab monotherapy (brand name Herceptin) is used to treat breast cancer patients with amplified or over-expressed human epidermal growth factor 2 (HER2+) in the body [1]. Researchers used Herceptin, which can also be combined with chemotherapy, to analyze the pathophysiologies of over 51 breast cancer cell lines with abnormalities in the genome which mirror over 145 primary breast cancer tumor types [2]. The process of cultivating human cells for research received impetus in 1951 when George Gey established the HeLa cell line named after Henrietta Lacks who had cervical carcinoma [3]. About eight years later, HeLa cells were established [4]. The first breast cancer cell line, referred to as BT-20, was established in the 1950′s and, since then, many other breast cancer cell lines have been established, such as MCF7 and MDA-MB-231 cells [5]. By analyzing these breast cancer cell lines, researchers can distinguish between different molecular aberrations to facilitate the identification of mechanisms that can help prevent or decrease the occurrence of breast cancer through therapeutical practices. Even though various breast cancer cell lines have been vital, challenges can occur, such as contamination due to poor technique or genetic drift due to over-passaging of breast cancer cells. Researchers have documented this instance of contamination from HeLa cells to other cultures that caused a large amount of ‘false cell lines’ to be produced [6].

Since then, many advancements have been made to categorize, illustrate, and utilize various breast cancer cell types for use by investigators. Notably, the MD Anderson series of various breast cancer cell lines that are known worldwide was established in 1973 at the Michigan Cancer Foundation [7]. T47D, MCF7, MDA-MB-231, and MDA-MB-157 are just a few breast cancer cell lines that have been used in vitro over the years to translate to in vivo studies. They have even found utility in studies of treatment for human breast cancer, e.g., Herceptin was found to predict the therapeutic response in many HER2+ cell lines such as SK-BR-3 [2]. Many have found that breast cancer cell lines are also modified by changes in gene expression that can be heritable from cell to cell, but do not modify DNA sequences [8]. This aspect of epigenetics has been shown through many different mechanisms such as chromatin remodeling, DNA methylation, effects from non-coding RNA, and histone modifications [9]. Some enzymes perform essential tasks within the epigenome to silence (e.g., DNA methyltransferase and histone deacetylase) or activate (e.g., histone acetyltransferase) genes in cells during development. Other methyltransferases can impact epigenetics by either altering the concentration of the methyl donors or creating a metabolic methylation sink. An example of this is the enzyme Nicotinamide N-methyltransferase (NNMT) which promotes epigenetic remodeling in breast cancer and affects all other NAD+ dependent enzymes such as poly-ADP ribose polymerases (PARPs) and sirtuins [10,11]. In addition, research has been performed using combinations of various dietary phytochemicals that induce inhibition of these silencing enzymes to analyze a decrease in the growth of cancerous cell lines [12].

There are remaining issues regarding cross-contamination when handling cell lines that could cause a crisis during experimentation [13]. This can occur, although proper sterilization and techniques are always helpful to combat inconsistencies. Cell line research can also translate to in vivo experiments when new connections are being constructed. For example, the gut microbiome has been analyzed to investigate any contribution to breast cancer prevention and treatment. Short-chain fatty acids such as sodium butyrate can be generated in the gut microbiome by digestion of food, and these compounds have enzyme-silencing properties that may contribute to a reduction in breast cancer development [14]. There has been a considerable amount of work performed with breast cancer cell lines, so much so that it can be difficult to find the origin of certain cell line types and the reasoning behind using a certain subtype in an experiment. Researchers need a foundational guide to organize the many categories of breast cancer cell lines, techniques to sustain them, and information on how to prevent contamination of these breast cancer cell lines. There have been publications that list the classifications of breast cancer cell lines and how they reflect heterogeneity from breast cancer [15]. However, there is a major gap in previous research to show a comprehensive overview of modern cell culturing techniques and example breast cancer cell lines that may apply when creating an experimental idea. 

The purpose of this review is to analyze and organize previous research performed on breast cancer cell lines to create a foundational starting point for investigators who are involved with cell-culturing experiments. If a certain cell line type is needed, researchers will be able to use this review for an overview of the origin of the cell line. Many investigators have made a large impact in this scientific field by classifying multiple types of breast cancer cell lines and describing the morphologies of each [16]. This review will extend such research by adding the techniques used to culture these categorized breast cancer cell types and how to maintain the lines without contamination. Having a foundational piece of work to guide researchers through the process of finding an ideal breast cancer cell line to work with during experimentation and key proteins or genes that they may be able to study is a valuable aspect that this review will bring to this research field. Even examples of techniques able to provide an ideal environment analogous to breast cancer tissue in vivo, cell-derived xenograft versus patient-derived xenograft, and how breast cancer cellular lines participate in creating pharmaceutical drugs that can execute functions in molecular pathways will be discussed. Also, understanding key components that are essential and may be detrimental to the growth of breast cancer cells will be explored. Through this review, many aspects of cell line research will be considered in detail. The goal is to categorize the various cell lines in such a way that investigators will be able to use this resource as a tool to help focus experimentation and further study.

## 2. Methods

Multiple empirical and review articles were accessed to investigate previous research on the history of breast cancer cell lines. PubMed and Google Scholar databases were searched using the keywords breast cancer, cell lines, molecular pathways, epigenetics, xenograft, cancer prevention, and cancer therapy. There was no meta-analysis, quantitative, or qualitative synthesis performed. Some references were published more than five years ago for the basis of grasping a history of cell line usage.

## 3. Results

### 3.1. Epigenetics and Breast Cancer

Analyses have been performed to discover the components of dietary phytochemicals such as epigallocatechin and sulforaphane that have been shown to decrease breast cancer risk [17]. These discoveries were originally performed with the use of in vitro cell lines and have significantly advanced the field. Analyses such as cell viability assay [(MTT(4,5-Dimethylthiazol-2-yl))] can reveal the number of cells that can survive from 3–7 days of treatment with these dietary phytochemicals that have been studied to show DNA methyltransferase (DNMT) and histone deacetylase (HDAC) inhibition. Gene-silencing enzymes such as DNMTs can attach a methyl group to a portion of DNA called a CpG (Cytosine–Phosphate–Guanine) island and prevent the transcription of that portion of DNA from being expressed. The other silencing enzyme HDAC has the role of removing an acetyl group from a histone structure, thus allowing the protein to be tightly coiled and unable to proceed with transcription [9]. If the dietary phytochemicals mentioned can inhibit the function of these silencing enzymes, more genes will undergo transcription and expression in the cells. Thus, knowledge of DNMT and HDAC inhibition properties of certain dietary phytochemicals is important to breast cancer cell line treatment and prevention in epigenetics.

Other experiments, such as western blot, PCR (Polymerase Chain Reaction), and enzymatic activity assays when a proper concentration of treatment (e.g., dietary phytochemicals) has been established, can be used to identify the optimal dosage to treat cancer cells and decrease their growth without toxicity to the control cell line of non-transformed cells. Studies have shown that some environmental factors can cause certain genes to be either silenced or expressed in the human body. As evidence of this, twins can have the same DNA but expressed differently based on the internal and external environment [18]. A common issue that often arises is the uncertainty of the exact number of polyphenols in the foods that humans consume to associate with the impacts they may have on various cancer types, specifically breast cancer [19]. This is why the use of breast cancer cell lines is so essential for this purpose; an exact number of dietary phytochemicals can be tested on cancerous cell lines to determine if there is a decrease in growth. Although research has accumulated over the years, there is still a limited number of studies on breast cancer cell lines [15]. In Table 1, various cell lines are shown that have been discovered to have either estrogen receptor (ER), progesterone receptor (PR), luminal–HER2, HER2, or triple-negative breast cancer basal-like/normal-like (TNBCA/B) properties along with the most beneficial medium in which they can be grown. The type of cancer cell line is also listed along with the morphology of the cancerous cell.

These breast cancer cell lines were chosen based on being commonly used and well-known with respect to various breast cancer cell line types. The *TP53* and protein status are also listed for the breast cancer cell lines. Multiple articles discuss *p53* mutations and how this gene is often called “the guardian of the genome”, as it can activate genes that play a role in cell cycle arrest, apoptosis, and DNA repair. Mutations within this gene may cause several aberrations to be multiplied and eventually produce proteins that are not supposed to be expressed in the cells of the body [41]. The *TP53* status of many breast cancer cell lines can be identified to highlight the actual mutation within the DNA binding domain. There has been much controversy over the years about the *TP53* status and how it can cause incorrect experimental interpretations. There is a collection of widely used cell lines from the NCI-60 panel that have the *TP53* mutant status collected for the ICGC/TCGA Pan-cancer Analysis of Whole Genomes databases [20]. This Pan-Cancer Project was established in 2006 and is an international collaboration for cancer genomics and the molecular characterization of over 20,000 primary cancers to match over 50 cancer types. By utilizing these mutation databases, analysis of *TP53* gene variants can be executed along with other important mutated genes in breast cancer research that are listed in Table 1.

### 3.2. Breast Cancer Cell Line Classification

**Figure 1:**
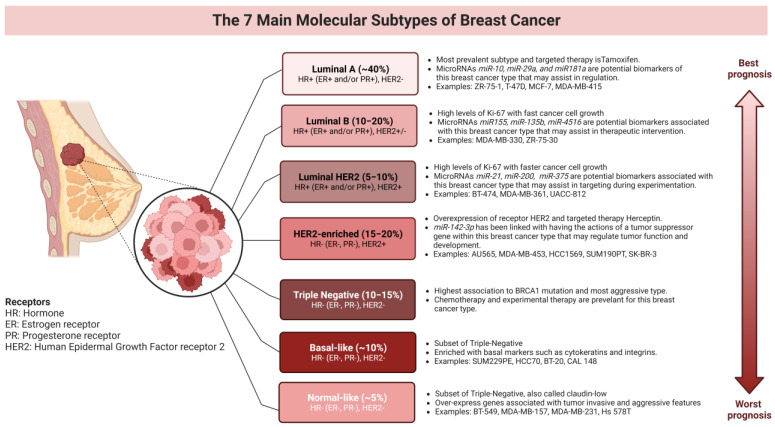
Types of breast cancer are grouped by Luminal A, Luminal B, HER2, and Triple−Negative. BC cell line examples; additional characteristics are also mentioned.

There are many ways in which to classify breast cancer cell lines, although the most common strategy that has been used over the past few decades entails immunohistochemistry (IHC) methods to recognize different hormone receptors such as ER, PR, and androgen (AR) [21]. IHC has been utilized to distinguish the expression through the phenotype of breast cancer cell lines with tyrosine kinase receptors such as HER2 and the Epidermal Growth Factor receptor (EGFR) [21]. Figure 1 shows a simplified schematic of breast cancer cell line types and their common characteristics according to the category. The first published work using IHC techniques dates to 1941, while the discovery of these influential hormone receptors to classify breast cancer cells dates back to 1940 (AR) and 1977 (ER and PR) [42,43]. Some investigators also include a normal-like subtype to classify about 2–8% of breast cancer that is also associated with Luminal A for being targeted with tamoxifen. Additionally, there is an intermediate subtype, Luminal HER2+, that is closely related to the HER2+ subtype owing to an overexpression of HER2 which has been linked to ER downregulation [16]. Immunohistochemistry is a technique that utilizes various types of antibodies to detect specific antigens in the tissue. The hormones estrogen, progesterone, and androgen have been recognized to be more prevalent in breast cancer tissue, and efforts have been made to target such a tissue through therapy or surgical approaches. By deriving the tissue from patients, breast cancer cell lines have been used to perform further tests that have become useful to treat different breast cancer types and reduce the risk of breast cancer in general [44,45,46]. These hormonal receptors can be found embedded in the phospholipid bilayer or free floating in the cytoplasm of many cells and function by allowing estrogen, progesterone, or androgen to bind. This then causes translocation to the nucleus or a cascade of enzymes to react and eventually silence or activate important genes that regulate transcription and produce genes that cause breast cancer to progress.

The origin of the name for most of the breast cancer cell lines discussed below is based on the scientists who derived the cell line, such as Zeida Rae for ZR-75-1 and Iafa Keydar who established T-47D at Tel Aviv University in the 47th petri dish [47]. Most of the other breast cancer cell line names originated from organizations such as the ‘M.D. Anderson Hospital and Tumor Institute’ for the MD Anderson-Metastatic Breast series, ‘Streamlined University of Michigan’ for the SUM series, ‘Hamon Cancer Center’ for HCC1569 and HCC70. Certain breast cancer cell lines were named after the number of cells that grew from a particular dish, such as the ‘BT’ series (BT-20, BT-549, BT-474), which includes the first breast cancer cell line derived and which was named by the researcher that isolated them, Etienne Y. Lasfargues, and the UACC-812 named by researchers from the ‘University of Arizona Cancer Center’ to create the University of Arizona Cell Culture series [47]. The MCF7 breast cancer cell line was named after the organization from which it was derived, the ‘Michigan Cancer Foundation’. The name AU565 originates from Adenocarcinoma Unknown 565 [47], while SK-BR-3 is named after the Memorial-Sloan Kettering Cancer Center, more specifically after the Sloan-Kettering Breast Cancer Cell Line 3 [47]. CAL148 breast cancer cell line owes its name to the Centre Antoine Lacassagne [47]. Hs 578T breast cancer cell line has a name that originates from *Homo sapiens* or Human sarcoma based on multiple sources [47]. 

#### 3.2.1. Hormonal Receptors

**Figure 2:**
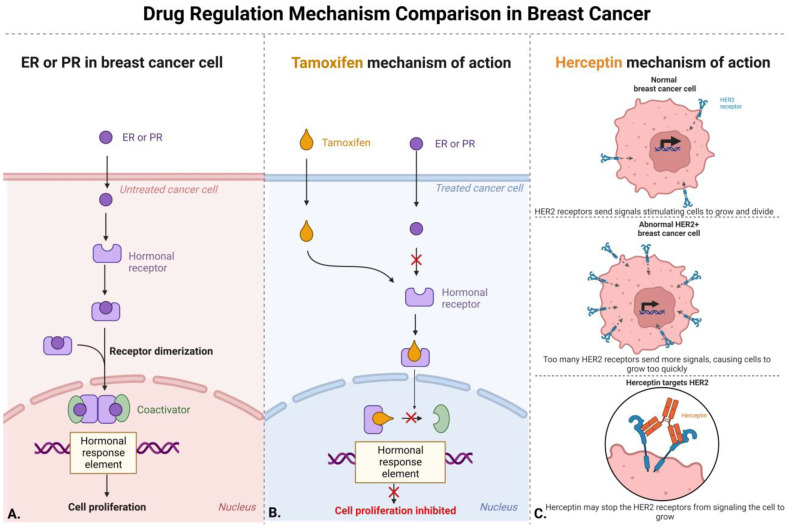
This schematic exemplifies the drug regulation mechanisms that occur in breast cancer when pharmaceutical drugs are administered. Part (**A**) shows an untreated cancer cell that receives either ER or PR ligands to bind to an intracellular hormonal receptor that will cause dimerization and increase breast cancer cell proliferation. Part (**B**) gives an example of tamoxifen affecting the molecular pathway by preventing the ER or PR ligand from binding to the hormonal receptor which inhibits breast cancer cell proliferation. The arrow with an red X represents the process not taking place due to binding of Tamoxifen during the pathway. Part (**C**) gives a schematic of Herceptin preventing HER2+ receptors from being able to express more signals in breast cancer cell.

Breast cancer may be caused by the mutation of multiple genes that are usually silenced or activated for expression. Many of these breast cancer cell lines are categorized by the hormonal receptors ER, PR, and AR that are derived from the breast cancer patient of origin. The absence or presence of the estrogen, progesterone, and androgen hormonal receptors can determine what kind of breast cancer cell line investigators would obtain for breast cancer experimentation. MDA-MB-453 breast cancer cell line, in particular, has a high level of AR, which has been shown to play a vital role in breast cancer pathogenesis [48]. AR expression is prevalent in breast cancer subtypes and has been found to be about 50% expressed in ER breast cancer types. The ER− or PR− status in breast cancer cell lines can determine if AR-targeted hormone therapy would be beneficial [49]. There has been treatment for ER through aromatase inhibition; however, this therapy technique alone may increase androgens, so usage of the hormonal therapy tamoxifen is mostly recommended to treat ER+ breast cancer in women and men [49]. As seen in Figure 2, the presence of ER+ in the patient’s breast cancer tissue can be treated with tamoxifen therapy, considering that ER+ may place breast cancer cell lines under the type Luminal A, the most prevalent type that also has low levels of Ki-67. One of the many important protein biomarkers for breast cancer is Ki-67 (derived from Kiel city and the number of the original clone 67). This biomarker has been studied to show an association with tumor cell proliferation and growth. When different types of tissue are tested through IHC monoclonal antibodies and tested for the expression of Ki-67, a prognosis may be made about the threshold and treatment available for patients [50]. Ki-67 was first studied by Scholzen to show that this protein is vital for the progression of the cell cycle, since it is present in each cell phase (G1, S, and G2, but not G0) [31,51,52].

PR+ breast cancer cell lines can be associated with the Luminal A type along with the Luminal B type that may be treated with tamoxifen hormonal therapy. The Luminal B subtype has a higher level of Ki-67 expression with faster cancer cell growth. In Figure 2, the HER2+ receptors are membrane-bound, showing that the targeted therapy for HER2+, Herceptin, will need the ability to enter the cell to cause homo- or heterodimerization through the cell signaling pathway [36]. HER2+ breast cancer cell type is also associated with receptor tyrosine kinases (RTKs), which play a role in cellular functions such as cell growth and survival. Other proteins that are RTKs are within the Erythoblastic oncogene B (ErbB) family, such as ErbB-1 (also referred to as epidermal growth factor receptor EGFR), ErbB-2 (also referred to as HER2), ErbB-3 (also referred to as HER3), and ErbB-4 (also referred to as HER4) [53]. These ErbB receptors are essential for the normal functioning of the body, but when present in excessive quantities cause dysregulation and can potentially lead to breast cancer [54]. Examples such as the ones reported above illustrate how vital these hormones and membrane receptors are for the overall advancement of breast cancer cell line research.

#### 3.2.2. Luminal Breast Cancer Cell Lines

ZR-75-1, T-47D, MCF7, and MDA-MB-415 all represent the Luminal A subtype of breast cancer, and all were established in the 1970s. The nomenclature for each of these breast cancer cell lines is unique and most have a mass structure according to Table 1. The ZR-75-1 breast cancer cell line was first derived from the isolated metastatic ascites of a 63-year-old white female patient and has been used to study different radioactive diagnostic agents that are used with PET (positron emission tomography) imaging, such as fluoroestradiol [22]. Being able to visualize ER+ breast cancer gene expression in ZR-75-1 cell lines was vital to further clinical applications. The T-47D breast cancer cell line was originally isolated from the metastatic pleural effusion from a 54-year-old female patient [55]. Studies have shown that T-47D is more susceptible to progesterone compared to the breast cancer cell line MCF7, progesterone being a hormone that is known to be prevalent in Luminal A breast cancer. The MCF7 breast cancer cell line was derived in the 1970s from a 69-year-old white female patient with metastatic pleural effusion and has since been one of the most well-known breast cancer cell lines [56]. Sources show that MCF7 has been searched the most (up to about 22,000 times depending on whether the hyphen is included) [57]. This ER+ breast cancer cell line has been used for 50 years and has many sub-clones, such as LM-MCF7, that have demonstrated high metastasis potential, downregulation of p27 (cyclin-dependent kinase inhibitor, also called KIP1, which is a tumor suppressor) expression, and upregulation of bcl-2 (b-cell lymphoma-2 is a regulator protein of cell death) protein expression when injected into severe combined immunodeficiency mice (SCID) [58]. Finally, Luminal A breast cancer cell line MDA-MB-415 was derived from a 38-year-old white female patient with pleural effusion at the metastatic site in 1975 [7]. MDA-MB-415 was used in a study to show that overexpression of the tumor suppressor p53 regulates apoptosis-inducing protein 1 (TP53AIP1) and can decrease cell viability in the breast cancer cell line [59].

All these Luminal A breast cancer cell line examples have in common a low percentage of Ki-67, meaning the recovery from breast cancer in the clinical setting is manageable. Some studies show the opposite of not having enough Ki-67 expression for Luminal A breast cancer, rendering the prognosis difficult without having that marker present [21]. Ki-67 was first discovered as an antigen in 1983 and has since been used as a proliferation marker for tumor growth in breast cancer cells [60]. Being able to analyze the expression of Ki-67 through all phases of the cell cycle, with the exception of G0, makes it an ideal tool to investigate the regulation of breast cancer growth and apoptosis [23]. There has been some controversy around the usefulness of the Ki-67 marker for Luminal A breast cancer prognosis based on the inconsistent cut-off range of 10–20% within the specimen for different research findings [61]. Immunohistochemistry staining of breast cancer tissue seems to be the primary tool for the identification of Ki-67 expression in breast cancer patients and is continuously being used for the prognosis of breast cancer progression status. Luminal A breast cancer subtypes have the advantage of being characterized by the presence of the ER+ receptor which can be manipulated in experiments involving the antiestrogen tamoxifen, the latter believed to be able to treat breast cancer long-term [62]. However, there are also disadvantages associated with the usage of Luminal A type breast cancer cell lines, such as the high levels of differentiation that can render the interpretation of the morphology difficult for various breast cancer cell line types within the category [16].

MDA-MB-330 and ZR-75-30 are the breast cancer cell lines of choice to represent the Luminal B subtype of breast cancer for this review. BT-474, MDA-MB-361, and UACC-812 are the breast cancer cell lines of choice that represent the Luminal-HER2 subtype of breast cancer cell lines for this review. Many of these Luminal B cell line types have a grape-like structure, as shown in Table 1. MDA-MB-330 was derived from a 43-year-old white female patient with metastatic pleural effusion in 1974 [7]. This breast cancer cell line is associated with invasive lobular carcinoma (ILC), rendering the transition from in vitro work to clinical therapy practices more specific and beneficial for patients with respect to treatment [26]. There has also been controversy with the ER+ status for this breast cancer cell line, hence why Table 1 shows +/− for ER in MDA-MB-330 cells [27]. ZR-75-30 is a breast cancer cell line derived from a 47-year-old black female patient in 1999 via metastatic ascites [28]. This breast cancer cell line is often associated with metastasis-associated proteins (MTA) MTA1 and MTA2 that depict overexpression or reduced expression to contribute to the metastasis of the ZR-75-30 breast cancer cell line [63]. Additionally, dietary compounds with epigenetic mechanisms such as berberine within *Coptis chinensis* have been shown to inhibit growth within the ZR-75-30 breast cancer cell line [64]. BT-474 was derived from a 60-year-old white female patient in 1978 who had invasive ductal breast carcinoma [65]. This breast cancer cell line has been used to improve the therapeutic efficiency against breast cancer with the use of the chemotherapy drug Hydroxyurea (HU) [66]. MDA-MB-361 was one of the nineteen cell lines derived at the M.D. Anderson Hospital and Tumor Institute in Houston, Texas. This breast cancer cell line was derived from metastases in the brain of a 40-year-old white female patient in 1975 [7]. The M.D. Anderson’s series has been influential in cancer discovery and prevention. For example, by knocking out the *MALAT1* (metastasis associated lung adenocarcinoma transcript 1) gene with the use of clustered regularly interspaced short palindromic repeats (CRISPR) editing in the MDA-MB-361 breast cancer cell line, decreased proliferation and increased apoptosis has been observed, a phenomenon which can be further studied in vivo in a clinical setting for breast cancer patients [67]. In 1986, UACC-812 was derived from a 42-year-old white female patient who had ductal carcinoma [32]. Some researchers have shown this breast cancer cell line as a HER2 subtype because only ErbB-2 is expressed in experiments completed via PCR, culture assays, or other methods [68].

The Ki-67 protein biomarker for proliferation in cancerous cells is high in Luminal B type breast cancers [51]. This means that the cell lines have a larger number of proliferating cells that cause the cancerous cells to divide more quickly [69]. This is not the only gene that can be used to characterize Luminal B type breast cancer. *BRCA1* was first discovered by Dr. Mary-Claire King in 1994 and the gene is associated with hereditary breast cancer [70]. *BRCA1*, along with the *BRCA2* gene which is more prevalent in male breast cancer, is a tumor suppressor that helps repair DNA double-strand breaks and decrease the amount of rapid cancerous growth in cells. Studies have shown that, when comparing Luminal type breast cancer to TNBC, *BRCA1* is equally downregulated in mRNA expression for Luminal type and TNBC [71]. This does not explain why luminal breast cancers have a better prognosis and are an easy target for certain hormonal therapies like tamoxifen. Another gene that is highly studied within different types of breast cancer is *TP53*, which has responsibilities in the endocrine response and resistance pathway [72]. Mutations within the *TP53* gene have shown that this tumor suppressor can be a great biological marker for therapeutic strategies in breast cancer treatment. The prevalence of certain genes in these breast cancer cells can be utilized to create different medications in clinical settings. As seen in Figure 2, there is a specific pathway that these pharmaceutical drugs take to be able to intercept the molecular mechanisms of breast cancer with hormonal receptors (ER and PR) or membrane receptors (HER2). However, much work remains to be done to maintain these breast cancer cell lines and ensure no genotypic variants affect the results being produced. This may be seen as a disadvantage associated with the utilization of Luminal B breast cancer cell line types, as the more invasive characteristics weaken the luminal phenotype that is associated with high expression of HER2+ expression which, in turn, downregulates the expression of ER+ [16].

#### 3.2.3. HER2+ Breast Cancer Cell Lines

MDA-MB-453, HCC1569, SUM190PT, AU565, and SK-BR-3 are all HER2+ breast cancer cell lines that do not have hormonal receptors for ER or PR. Grape-like structures and mass morphology are common within this type of breast cancer. MDA-MB-453 was first derived in 1976 from a 48-year-old white female patient with metastatic pericardial effusion [7]. This first example of HER2+ breast cancer cell lines has been a vital tool for the identification of molecular mechanisms with certain compounds used to treat cancer, such as hirsuteine [73]. Hirsuteine is an alkaloid that can be extracted from *Uncaria rhynchophylla* to treat different diseases, but the mechanism of downregulating BCL-2 and promoting apoptosis was not discovered until the use of the MDA-MB-453 cell line. MDA-MB-453 cells are an androgen-responsive breast carcinoma cell line with high-level AR expression. The HCC1569 breast cancer cell line was derived from a 70-year-old black female patient by isolating the mammary gland with metaplastic carcinoma in 1995 [74]. Since then, this breast cancer cell line has been used in the analysis of synergistic effects within the combination of PI3K (plasma membrane-associated lipid kinases) inhibitors and Herceptin to develop a less toxic implementation protocol for cancer patient treatment [75]. Unfortunately, researchers have yet to find any significant alteration within PTEN (phosphatase and tendon homolog that is a tumor suppressor) through the combination of treatment from PI3K and Herceptin applied to the HCC1569 breast cancer cell line [75], so more studies are required to find better concentrations or methodologies. 

SUM190PT was first derived from a 40-year-old woman’s primary tumor in 1996 and has been shown to lack ER and PR [76]. The SUM breast cancer cell line was established in the 1990s and has such a following that the SUM breast cancer knowledge base (SLKBase) was created for researchers to comment and post about different experiences, research attributes, and findings concerning this cell line and others [76]. SUM190PT has been used to observe the effectiveness of histone deacetylase (HDAC) inhibitors in creating new therapy treatments for cancer patients. Findings have shown that the HDAC inhibitor CG-1521 does induce apoptosis, although it targeted tubulin (non-histone protein used for spindle assembly in the cell). Therefore, more work needs to be performed to ensure the safety of CG-1521 for treatment purposes [77]. AU565 was first derived in 1970 from a 43-year-old white female patient with breast adenocarcinoma from the metastatic pleural effusion [78]. This breast cancer cell line is known to overexpress the ErbB2 protein [79]. The SK-BR-3 breast cancer cell line was derived in the same year as AU565, from the same 43-year-old white female through the pleural effusion metastasis that has similar characteristics of overexpression of the ErbB-2 gene product. Investigators discovered that SK-BR-3 was stimulated by the activation of the ninth EGF-like domain that contained GST (glutathione *S*-transferase) fusion proteins to indicate that no other ErbB2 ligand was derived from that specific EGF-like domain. There have also been studies conducted using flaxseed lignans in combination with pharmaceutical drugs that caused a decrease in cell viability in SK-BR-3, suggesting a potential improvement in chemotherapeutic applications in the clinical setting [80].

The ErbB receptor family is important for this type of breast cancer cell line, since HER2 is included as a receptor. A common characteristic of HER2+ breast cancer is that the prognosis is poor and the ability to treat this type of breast cancer is limited. Studies have been conducted with genes such as *EGF* and *hMena* (cytoskeleton regulatory protein) to analyze the phosphorylation and cell proliferation of HER2+ breast cancer cells [81]. This study did show that the downstream results of the knockout of *hMena* affecting activity of EGF in the cell may be a great attribute to identify new methods for the prognosis in breast cancer patients. An advantage of working with HER2+ breast cancer cell lines is that the HER2+ receptor allows certain drug targets to attach when conducting experimentation, e.g., Herceptin. The translation from in vitro to in vivo formed poorly differentiated tumors in immunocompromised mice with HER2+ SK-BR-3 cell line, so this may be a disadvantage [16].

#### 3.2.4. Triple-Negative Breast Cancer Cell Lines

**Figure 3:**
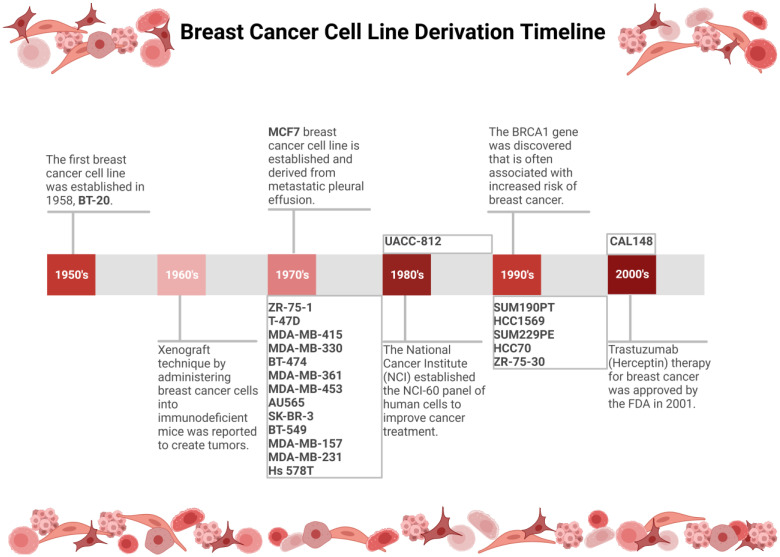
Time of breast cancer cell line derivation along with additional novel findings from breast cancer cell line research. Abbreviations for each breast cancer cell line origin can be found in Section 3.2.

SUM229PE, BT-549, HCC70, BT-20, CAL148, MDA-MB-157, MDA-MB-231, and Hs 578T are all breast cancer cell lines that have been classified as triple-negative breast cancer (TNBC) [16,24,40]. Figure 3 illustrates a timeline of the cell line derivations along with key elements that have taken place in breast cancer cell line research. TNBC cell lines lack ER, PR, and HER2 and have been known to be the most aggressive/worst prognosis breast cancer type [16]. This breast cancer category is also known to be most common in black women who have a familial history of cancer [82]. Stellate is the most common structure among TNBC cells, along with a few that show round, mass, and spindle structures, as shown in Table 1. SUM229PE is a breast cancer cell line that was retrieved in 1996 from the pleural effusion fluid of a female patient [76]. This breast cancer cell line has also been used to study resistance to inhibitors such as MEK1/2 (mitogen-activated protein kinase 1/2) and as a control to test the vulnerabilities in drugs that may affect heterogeneity amongst the different cancerous cell types [39]. In 1978, BT-549 was derived from a 72-year-old white female patient by isolating the papillary of metastasized portions of the lymph node [83]. This breast cancer cell line showed downregulation of HDAC7 and established the possibility of this gene being a tumor suppressor in TNBC [84]. HCC70 was first derived from a 49-year-old black female patient in 1992 by isolating a primary ductal carcinoma [75]. This TNBC cell line has been used to study the effectiveness of cancer treatment drug conjugates on breast cancer cells, such as the peptide TH1902 for docetaxel [85]. BT-20 was the first breast cancer cell line to be derived in 1958 from a 74-year-old white female patient by isolating the tumor within her breast [5]. This hallmark breast cancer cell line has been used in studies to show alterations in mitochondria due to the function of key elements of the mTOR (mammalian target of rapalycin) pathway [86]. The CAL148 breast cancer cell line was derived from a 58-year-old French female patient with pleural effusion in 1994 [87]. In 2019, CAL148 was used to discover if two drugs, palbociclib and MLN0128, could work synergistically to inhibit cell proliferation, with results revealing that further investigation in the clinical setting would be beneficial [88]. MDA-MB-157 was first derived in 1972 from a 44-year-old black female patient with metastatic breast cancer and pleural effusion [7]. Both MDA-MB-157 and MDA-MB-231 have been used to study the effects of the HDAC inhibitor Panobinostat. Research has shown that this HDAC inhibitor is toxic to TNBC and could be a potential tool for treatment [89]. In 1973, MDA-MB-231 was derived from a 51-year-old white female patient by isolating the mammary gland of an adenocarcinoma via metastatic pleural effusion [7]. This TNBC cell line is known to be very aggressive and is associated with a poor diagnosis. The Hs 578T cell was first derived in 1977 from the breast of a 74-year-old white female patient [90]. This cell line has since been used as a tool to evaluate HMGA1 (high mobility group A) protein expression, as it relates to mitochondrial mutation in cancer cell research [91].

There has been controversy over the years concerning the MDA-MB-435 cell line and its derived origin. Investigators have shown that the MDA-MB-435 cell line was derived from a 31-year-old white female patient in 1976 with metastatic, ductal adenocarcinoma of the breast [92]. However, studies through gene analysis have depicted the clustering of this cell line with melanoma-origin cell lines. This discovery was made in 2000 by DNA microarray analysis and, following debate in 2007, the MDA-MB-435 cell line was determined to have originated from melanoma [93,94]. Even though this issue has been settled, there have still been scientific articles published that categorize the cell line as originating from breast cancer.

TNBC constitutes 10% to 15% of all breast cancers, and an important component to distinguish the presence of TNBC is retinoblastoma (RB1) status. RB1 is a tumor suppressor that is not found in TNBC and is currently being studied to understand its effects within specific therapies [95]. Research has shown that the presence of RB1 in TNBC lines is more sensitive to gamma radiation and that few RB1 are present in TNBC. All TNBC cell lines discussed above are examples from many studies aiming to reach the goal of cancer cure and prevention. More in vivo work is necessary as well as in the clinical setting to ensure proper verification of methodologies and techniques and prevent and eventually cure cancer. In vitro studies have been essential to understand breast cancer and will continue to inform and stimulate future research. TNBC cells also have subsections that are basal-like for enrichment with basal markers and claudin-low, which is associated with genes that are tumor-invasive and aggressive [16]. An advantage of TNBC cell line use is the characterization with *BRCA1* gene mutation for the basal-like subsection that can be analyzed with cell lines for the translation to clinical settings. A disadvantage of utilizing TNBC cell lines is the lack of receptors that may show relatability to immunotherapy that is done in the clinical setting [96]. However, dietary compounds have been shown to restore ER+ gene expression in TBNC through epigenetic mechanisms that may be applicable to chemotherapeutic approaches with additional findings [97].

### 3.3. Common Requirements, Techniques, and Approaches to Cell Culture Maintenace

#### 3.3.1. Medium Choice and Control Cell Lines

These breast cancer cell lines must have a mixture of components for each medium type to ensure the proper nutrients are available to support cell growth and maintain cells viable for experimental studies. In Table 2, the components for each of the breast cancer cell lines from Table 1 are outlined. These are examples for each breast cancer cell line and the addition of antibiotics is based on laboratory preference. Some investigators also recommend the addition of a DMEM high-glucose medium instead of a DMEM normal-glucose medium for cell culture components to ensure higher maintenance efficiency in breast cancer cell lines [98]. Investigators can choose various medium types and add the necessary nutrients for the breast cancer cell lines being utilized. Common media of choice are Human Plasma-Like Medium (HPLM), Minimal Essential Medium (MEM), Iscove’s Modified Dulbecco’s Medium (IMDM), Dulbecco’s modified Eagles Medium (DMEM, high glucose), DMEM/F-12 nutrient media, Ham’s F-12 Nutrient Medium, and Roswell Park Memorial Institute 1640 Medium (RPMI 1640, low glucose) [99]. Often, Fetal Bovine Serum (FBS), a growth supplement, is used to promote growth through proteins and growth factors in a cell culture environment. Antibiotics such as streptomycin, amphotericin B, and penicillin are used to prevent cell wall synthesis and interfere with cell permeability and bacterial development in cell cultures. On the other hand, antibiotics such as penicillin and streptomycin have been shown to also alter gene expression, cell regulation, and drug response. This is an aspect that scientists must consider when designing an experiment. In addition to the medium of choice, certain additives can be placed within the medium to ensure proper breast cancer cell line maintenance, such as pyruvate that stabilizes the hypoxia-inducible factors in TNBC cells [100]. The environment within which all these breast cancer cell lines must remain to grow is a laboratory-validated incubator at 37 °C. While a 5–10% CO_2_ and air mixture is used in association with most available culture media, Leibovitz’s L-15 medium (used for the UACC-812 cell line) is detrimental to cell cultivation in a CO_2_ and air mixture environment [32].

The MCF10A cell line is non-cancerous and has different strains that have been used by many investigators. Breast cancer cell line maintenance is vital for the success of experimental trials, leading, hopefully, to clinical trials. The presence of control cell lines is a requirement to ensure the data acquired are reliable and accurate. At least two, preferably three, cell lines and one control cell line is what most consider to be the optimal number of cell lines to obtain reliable data. For example, an investigator used [ER− PR−] MDA-MB-231 and ER+ MCF7 breast cancer cell lines along with MCF10A control cells in their study for combinatorial epigenetic mechanisms of sulforaphane, genistein, and sodium butyrate in breast cancer inhibition [12]. The MCF10 cell line was derived in 1990 by Soule and colleagues as the first non-transformed, human mammary epithelial cell line derived from normal breast tissue [112]. Multiple sublines including MCF10AneoT, MCF10AT, MCF10DCIS, and MCF10CA1 have been used as excellent models to help analyze and classify many breast cancer cell line types. Although MCF10 cell lines are widely known and the most commonly used benign proliferative breast cancer tissue-derived cell line, there have been studies that show MCF10A cells may not represent luminal, basal, and normal cells, phenotypically, when placed in different culture systems (2D versus 3D) [113]. 

The use of human mammary epithelial cells (hMECs) has also played a vital role in breast cancer cell line research, representing another control cell line for the comparison with breast cancer cell line progression and development [114]. hMECs are normal epithelial cells that have been utilized to monitor, in vitro, the early stages of tumorigenesis along with the ability to reprogram to a previous state (neoplastic) [115]. There have been issues with the short, allotted time for passages during the cell culture process. hMECs are already destroyed embryos from stillbirths that are difficult to grow in a lab setting. Based on research conducted previously, investigators have found that the passage time is limited to 5–8 times before the cell line is unable to be chosen as a control cell line for breast cancer research. On the other hand, MCF10A cell lines have been utilized within so many sublines and, even though the growth phase is slower than most breast cancer cells, the cell line is an optimal choice for breast cancer cell line work. 

#### 3.3.2. Cellular Techniques and Morphology

The discovery of histological differences in origin can be stated to have started in 1906 when Histology was recognized as a biomedical discipline [98]. Advances in microscope technology have enabled investigators to observe inside organs, tissues, and even cells. The ability to distinguish between different cell types was not possible until the invention of the microscope in the 1600s and. Since then, the evolution of histology has hastened. The classification of different shapes for breast cancer types in Table 1 was established using the first 3D models of cell cultures in microenvironments in 2007 [77]. The grape-like morphology of breast cancer cell types is mostly associated with Luminal B breast cancer, but AU565, which is an example of triple-negative breast cancer, ZR-75-1, associated with Luminal A, and SK-BR-3, which is an example of HER2+, have also been classified as exhibiting a grape-like morphology. 

**Figure 4:**
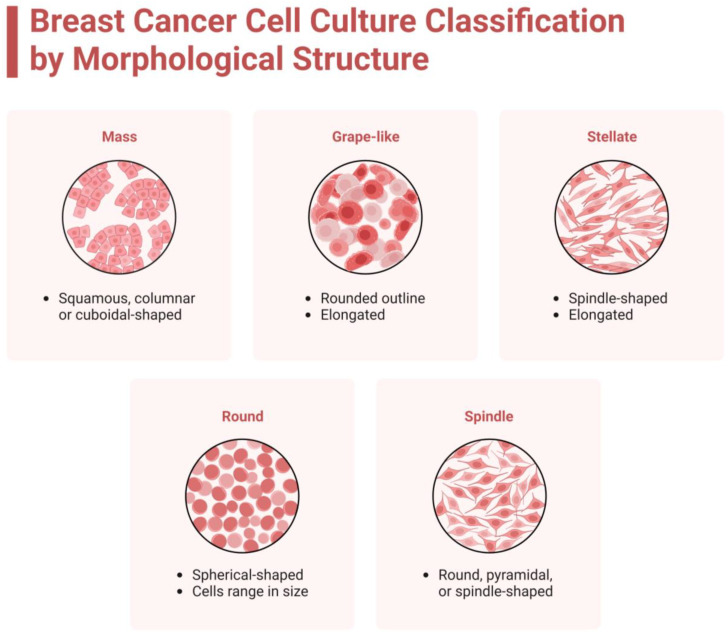
Types of morphology to group the Luminal A, Luminal B, Luminal-HER2, HER2, TNBC, and other cancerous cell lines. Each breast cancer cell line morphology is explained under Section 3.2.2, Section 3.2.3, Section 3.2.4 and Section 3.3.2.

The round morphology is associated with MDA-MB-415 and CAL148 breast cancer cell lines. These breast cancer cell types are from two different categories (Luminal A and TNBC, respectively), but there is a common protein that is associated with round morphology breast cancer cell types. Moderate levels of ErbB-2 are required for the formation of round morphology cell lines, while higher expression levels are required for HER2+ cell lines [66]. Mass morphology is very diverse and incorporates breast cancer cell types from all four categories. Mass morphology breast cancer cell types include T-47D, MCF7, BT-474, HCC1569, and HCC70, all of which have the highest level of proton ErbB-2 expression from western blot analysis [66]. Stellate morphology is characteristic of all TNBC cell lines, including BT-549, BT-20, MDA-MB-157, MDA-MB-231, and Hs 578T. MDA-MB-435 is also classified as having stellate morphology despite being a melanoma-derived cell line [88]. The morphology of each breast cancer cell line has been described, including mass, grape-like, stellate, round, and spindle which are depicted in Figure 4.

**Figure 5:**
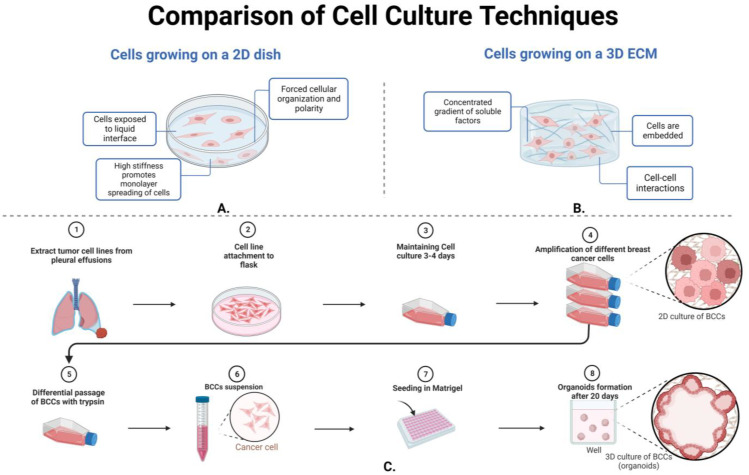
There are key differences between the utilization of 2D cell cultures and 3D cell cultures. Part (**A**) shows that breast cancer cells grown on a 2D dish have a forced cellular organization and polarity, high stiffness to promote monolayer of spreading cells, and no cell–cell interaction. Part (**B**) illustrates 3D cell culturing techniques to embed breast cancer cells in ECM, incorporate cell–cell interactions, and form a more complex culturing system. Part (**C**) illustrates most breast cancer cell line derivation from pleural effusions to plating in a 2D culture flask for amplification of the differentiated cells. While organoid formation can then be formed after proper breast cancer proliferation and embedding in the proper ECM material, see Section 3.3.2 for more information.

The process of creating 2D versus 3D cell culture models is summarized in Figure 5. Maintaining 2D cell cultures has the advantages of being cost-effective and convenient while allowing diffusion of soluble factors into the media. Disadvantages of 2D cell culturing include reduced cell-to-cell interactions and the less translatable models [116]. Three-dimensional culturing has been expanded with an additional technique of co-culturing between the MCF7 breast cancer cell line and MRC-5 fibroblast spheroids to study the many mechanisms that contribute to a 3D environment [117]. The utilization of 3D cell culture techniques allows a more accurate representation for translation to in vivo studies, increases cell-to-cell ECM interactions, and the matrix fibrils can restrict cell spreading [118]. The disadvantages of using 3D culture include the handling of a more complex culture system, as seen in Figure 5, which includes ECM layers to incorporate an aspect of the basement membrane (matrigel, collagen, polydimethylsiloxane, and laminin) [119]. Breast cancer cell lines that have been utilized as spheroid models cultured in a 3D system include, but are not limited to, ZR-75-1, T-47D, MCF7, MDA-MB-415, BT-474, MDA-MB-361, UACC-812, MDA-MB-453, HCC1569, AU565, BT-540, HCC70, BT-20, MDA-MB-231, and Hs578T cells [24]. Additionally, cellular techniques can be utilized to maintain various breast cancer cell line types. 

## 4. Additional Concepts Involving Breast Cancer Cell Line Research 

### 4.1. Cross-Contamination

Cross-contamination of breast cancer cells can occur through multiple means such as culturing techniques and mislabeling of containers [120]. HeLa cells were the first to be developed in 1952 from glandular cancer of the cervix [6]. The HeLa cell confusion caused a catastrophe in many laboratories over 60 years ago, but there are still breast cancer cell lines in use that derive from that very discovery [3,121]. Sterilization of the area within the Biosafety Cabinet (BSC) and gloves when handling breast cancer cells with 70% ethanol is essential to keep contamination at a minimum. Studies have shown that benzalkonium chloride with corrosive inhibition and distilled water in wet conditions would be the optimal combination for the sterilization of the BSC area with respect to a specific bacterium [122]. Considering that benzalkonium chloride with corrosive inhibition may not be the optimal option for most laboratories, 70% ethanol is a common choice for use when sterilizing surfaces. Freezing cells for future use can ensure the longevity of a breast cancer cell line, but care must be taken when labeling and opening the cryovial for the re-suspension of cell lines. If a 37 °C water bath is not properly maintained to thaw frozen cell lines for <1 min, this can cause contamination. Removing cell lines from liquid nitrogen storage and placing them directly in the water bath is essential to maintain cell viability. If placed on ice temporarily, breast cancer cells can thaw and die before being plated. UV radiation is a great method before and after cell culturing to maintain a sterile environment and not cause any cross-contamination. Investigators have discovered that UV radiation can cause DNA methylation alteration in cells; therefore, proper protocols should be in place in a laboratory environment to prevent this from influencing cellular morphology [123].

### 4.2. Genetic Mutations

Studies have shown that breast cancer cell lines may have more mutations than the tumor from which they are derived, and this raises concerns for researchers who are interested in translating conclusions found from breast cancer cell line studies [16]. History has shown that an increase in mutated BRCA1 or BRCA2 can put patients at higher risk of developing cancer, but the origin of many of these breast cancer cell lines studied may have mutated. An example of a breast cancer cell line that is widespread and has been shown to have diverse genetic uncertainties through multiple analyses is MCF7. This breast cancer cell line has been shown to react differently to drugs used over a length of time from the various subculturing processes to the freezing and thawing of the cells [124]. Prevention of genetic mutations of breast cancer cell lines is possible, with helpful techniques including maintenance of a sterile environment when subculturing, minimization of the freeze–thaw process, and documentation of the cell line passage number to prevent overpassaging [100]. Additional genetic mutations that have been utilized and used as tools to target breast cancer are cyclin-dependent kinase inhibitor 2A (*CDKN2A*), phosphatidylinositol 3-kinase Catalytic Subunit Alpha (*PIK3CA*), phosphate and tensin homolog (*PTEN*), and *TP53*, which is a tumor suppressor protein p53 that was previously mentioned [125,126,127,128]. Another novel molecular biomarker that has been influential in oncological decision-making is programmed death ligand 1 (*PD-L1*), which has been shown to have intrinsic capabilities concerning triple-negative breast cancer cells. The knockdown of this immunosuppressive protein has been shown to decrease cell proliferation and tumor growth in the model organism chosen for the experiment [129]. Examples such as these illustrate the essential role breast cancer cell lines play in the development of therapeutic techniques for breast cancer prevention and therapy. There are numerous genes that can be either overexpressed or underexpressed in breast cancer cell lines, and a few of those that have been studied are mentioned in Table 1, e.g., cadherin 1 (*CDH1*) and FA complementation group A (*FANCA*) that has been shown to be associated with lobular breast cancer [130,131,132]. Spen family transcriptional repressor (*SPEN*) has been associated with poor prognosis of breast cancer and is involved with chromatin remodeling [130,133]. Catenin alpha 1 (*CTNNA1*) and mediator complex subunit 12 (*MED12*) have been shown to associate with breast cancer [130,131,134].

### 4.3. Cell-Derived Xenograft and Gut Microbiota in Breast Cancer

**Figure 6:**
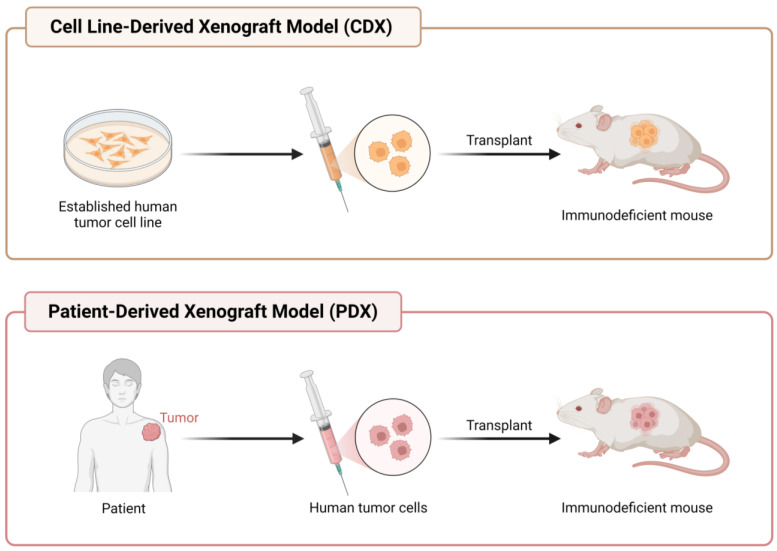
Comparison of the Cell Line-Derived Xenograft (CDX) model process versus Patient-Dervied Xenograft (PDX) model process.

Utilization of patients with breast cancer cells has been employed with respect to many techniques to diagnose and investigate various methods for better treatment of the disease. The first xenograft technique was implemented in 1962 from human breast cancer to an immunodeficient mouse [135]. The technique of patient-derived xenografts (PDXs) has been conducted by numerous institutions for therapeutic and clinical trials [136]. This process involves the removal of cells from a patient with a known status of breast cancer and the injection of such cells into an immunodeficient mouse in a specific area that will show the growth of a tumor for analysis, as seen in Figure 6. The exact location of injection could be, for example, intraperitoneally or orthotopically, although it depends on the the type of cancer. Injection of breast cancer cells into the mammary fat pad of immunocompromised mice is the most common example of PDXs. Many strains of immunodeficient mice have been utilized since the discovery of the ‘nude’ mice model in 1962 by Grist [137]. An interesting concept that has been discovered is that the difference between PDXs and cell-derived xenografts (CDXs) may not be as obvious according to pathologists viewing histology slides [138]. Viewing immunohistological quality control (QC) slides is a daily routine in pathology; therefore, utilization of CDXs instead of PDXs may be beneficial if there is no clear difference. 

One connection that has been investigated but needs further investigation is the relationship between breast cancer and the gut microbiota environment. Studies have established that as a result of the introduction of (GE) genistein into mice diet, there were microbial alterations in members of the family Lachnospiraceae and Ruminococcaceae [139]. No significant difference in microbiota composition was found between pre-chemotherapy and post-chemotherapy fecal samples of breast cancer patients, although introduction of GE into their diet did induce epigenetic changes resulting in reduced tumor size and increased tumor latency. The use of known epigenetic factors from dietary compounds that can be included into the diet of immunocompromised mice may become vital for breast cancer research. The fact that the mice used in the pilot study were humanized mice and not injected directly with breast cancer cells shows how the heterogeneity of breast cancer cells translated appropriately. Utilization of CDXs over PDXs could uncover novel findings in breast cancer research and prevention, but the data to analyze translatable evidence that CDXs can be applied to human clinical trials are still being gathered and investigated. The Luminal A breast cancer cell lines MCF7 and T-47D have been frequently used to inoculate mice into xenograft models for further examination because of the presence of estrogen [55]. However, Luminal A or Luminal B breast cancer cells engrafted from PDXs are difficult to grow and maintain due to ER+ tumors [140]. Investigators have used HER2+ breast cancer cell lines such as MDA-MB-453 to discover metastasis ability through intravenous injection (IVI) for CDX models [141]. The TNBC cell line MDA-MB-231 has been utilized with CDXs to investigate a decrease in tumor growth and induce G1 cell cycle arrest when using targeting agents [142].

Researchers have analyzed the immunohistological stains for β-catenin, Ki-67, and E-cadherin in human cancer types versus cell-derived xenografts. The expression of β-catenin in tissue can result from aberrations in the Wingless-related integration site (Wnt) signaling pathway. Levels of Ki-67 in breast cancer tissue have been used as a biomarker protein for cell proliferation in breast cancer. E-cadherin expression in breast cancer cells can distinguish invasive ductal or lobular cancer. Being able to investigate these protein markers as they relate to the gut microbiome can show how estrogen in breast cancer reacts in the body. Utilization of CDXs may lower costs for experimentation and develop connections that are lacking in breast cancer research. Studies have uncovered phenotypical similarities that suggest that CDXs can play a role in further investigation of tumor budding in colorectal cancer [143]. Realizing how essential CDXs have been used in many cancer types to facilitate deeper understanding is important. On the other hand, studies have shown that six breast cancer cell lines (ER+:[UCD4, UCD12, and UCD65] and ER−:[UCD46, UCD115, and UCD178]) have been created from PDXs to increase the overall ER+ number of cell lines within the archive to manipulate for breast cancer research [144]. These findings may bridge the gap in recognizing the connection between breast cancer and the gut microbiota environment.

## 5. Conclusions

Breast cancer cell lines have been utilized for over 50 years to establish prognosis, protein biomarkers, morphological differences, and genetic mutations. There is much to be discovered still, but, through this review, a researcher can take advantage of the established knowledge that has been produced. By categorizing breast cancer cell lines into Luminal A, Luminal B, Luminal-HER2, HER2+, and TNBC, the investigator can be very precise when analyzing data and creating experiments to make novel advancements in science. By utilizing the unique techniques described, such as 2D versus 3D subculturing, xenograft experimentation, and, possibly, the application of various dietary compound concentrations to analyze epigenetic regulation within breast cancer cell line growth inhibition, investigators may discover creative applications that can be practiced through analyzing the breast cancer cell lines discussed in this review. Being able to understand and appreciate the components of breast cancer cell line types that include, but are not limited to, Ki-67 expression level and various genetic mutation statuses, the investigator may carefully analyze and choose the correct cell line that can coincide with their field of expertise. This review is intended to assist the researcher when creating new ideas, and can be used as a guidance resource when additional background information can be useful for the origin of various breast cancer cell types.

## Figures and Tables

**Table 1:** Breast cancer cell line classification.

Cell Line	^1^ ER	PR	HER2	Type	Protein Status	TP53 Status	Additional Mutated Genes	Medium	Morphology	References
ZR-75-1	+	+/−	−	Luminal A	Low Ki-67	Wild type	PTEN	RPMI	Grape-like	[16,20,21,22,23,24,25]
T-47D	+	+	−	Luminal A	Low Ki-67	L194F	PIK3CA, SPEN	RPMI, DMEM	Mass	[16,20,22,24,25]
MCF7	+	+	−	Luminal A	Low Ki-67	Wild type	PIK3CA	RPMI, DMEM	Mass	[16,20,21,22,23,24,25]
MDA-MB-415	+	+/−	−	Luminal A	Low Ki-67	Y236C	MAP2K4, PTEN	DMEM	Round	[7,16,20,22,24,25]
MDA-MB-330	+/−	−	+	Luminal B	High Ki-67	Y220C	CTNNA1	RPMI	Grape-like	[7,16,22,25,26,27]
ZR-75-30	+	−	+	Luminal B	High Ki-67	Wild type	BRCA2, AKT1	RPMI	Grape-like	[16,25,28,29]
BT-474	+/−	+	+	Luminal HER2	High Ki-67	E285K	PIK3CA, BRCA2	RPMI	Mass	[16,20,21,22,24,25]
MDA-MB-361	+	+/−	+	Luminal HER2	High Ki-67	E56 *	PIK3CA, SPEN	RPMI, DMEM	Grape-like	[7,16,22,24,25,30]
UACC-812	+/−	+/−	+	Luminal HER2	High Ki-67	Wild type	BRCA1	L-15, RPMI, DMEM	Grape-like	[16,24,25,30,31,32,33]
AU565	−	−	+	HER2+	High Ki-67	R175H	CDH1	RPMI	Grape-like	[16,21,24,25,30]
MDA-MB-453	−	−	+	HER2+	High Ki-67	T382S	BRCA2, PTEN, PIK3CA	RPMI, DMEM	Grape-like	[7,16,20,21,24,25]
HCC1569	−	−	+	HER2+	High Ki-67	E294 *	PTEN, PIK3CA	RPMI	Mass	[20,24,25,34,35]
SUM190PT	−	−	+	HER2+	High Ki-67	Q317 *	PIK3CA	Ham’s F12	Mass	[2,12,16,25,36]
SK-BR-3	−	−	+	HER2+	High Ki-67	R175H	CDK2NA	DMEM, McCoys	Grape-like	[16,20,21,24,25]
HCC70	−	−	−	TNBCA	High Ki-67	R248Q	FANCA, PIK3CA	RPMI	Mass	[16,20,24,25]
BT-20	−	−	−	TNBCA	High Ki-67	K132Q	RB1, PIK3CA	EMEM, RPMI, DMEM	Stellate	[16,20,21,25,37,38]
CAL148	−	−	−	TNBCA	High Ki-67	E224K	RB1, PTEN, PIK3CA	DMEM	Round	[16,25,30,38]
SUM229PE	−	−	−	TNBCA	High Ki-67	R273C	CDK2NA, PIK3CA	RPMI, Ham’s F12	Spindle	[2,16,25,30,37,39]
BT-549	−	−	−	TNBCB	High Ki-67	R249S	RB1, PTEN	RPMI	Stellate	[16,20,24,25]
MDA-MB-157	−	−	−	TNBCB	High Ki-67	A88fs	MED12, SPEN	RPMI, DMEM	Stellate	[7,16,20,25,40]
MDA-MB-231	−	−	−	TNBCB	High Ki-67	R280K	BRAF, TERT, KRAS	RPMI, DMEM	Stellate	[7,16,20,21,24,25]
Hs 578T	−	−	−	TNBCB	High Ki-67	V157F	PIK3CA, MED12, CDKN2A	RPMI, DMEM	Stellate	[16,24,25,30]
MDA-MB-435	−	−	−	MEL	High Ki-67	G266E	BRAF, CDKN2A	L15, RPMI, DMEM	Spindle	[16,20,25]

^1^ ER (estrogen receptor), PR (progesterone receptor), HER2 (human epidermal growth factor receptor 2), and MEL (Melanoma). Medium is the culture media that the breast cancer cells can be grown in, but this is not limited to what is shown. Options for media include EMEM (Eagle’s Minimum Essential Medium), L-15 (Leibovitz’s L-15 Medium), RPMI (Roswell Park Memorial Institute-1640 Medium), DMEM (Dulbecco’s Modified Eagle Medium), McCoys (McCoys 5a Medium) and Ham’s F12 (Ham’s F-12 (Kaighn’s) Medium). All media have nutrients added to ensure proper cell viability (see Section 3.3.1 for more information). +/− represents the positive or negative receptor that the breast cancer cell line is classified to have The type consists of the receptors that are present or not present within the breast cancer cell line such as Luminal A, Luminal B, Luminal HER2, HER2+, and Triple-Negative Breast Cancer (TNBC) that can be further divided into Basal-like (TNBCA) and Normal-like (TNBCB). Protein status consists of the level of Ki-67 proliferation index of the breast cancer cell line type. *TP53* status indicates the protein sequence mutation for each breast cancer cell line listed. Some of the amino acid alterations have an asterisk symbol “*” that indicates a premature stop codon that is produced from the *TP53* mutation. Wild type indicates that the breast cancer cell line does not have a mutation within the *TP53* gene. Morphology consists of stellate, mass, grape-like, spindle, and round.

**Table 2:** Optional medium choice for breast cancer cell line maintenance.

Breast Cancer Cell Line	Foundational Media	Supplement Additives	References
ZR-75-1	RPMI	10% FBS, 10 mL penicillin and streptomycin	[56]
T-47D	RPMI/DMEM	100% FBS, 100 IU/mL penicillin, 100 μg/mL streptomycin	[55,101]
MCF7	RPMI/DMEM	10% FBS, 100 IU/mL penicillin, 100 μg/mL streptomycin	[55,101]
MDA-MB-415	DMEM	10% FBS, 100 U/mL streptomycin and penicillin	[60]
MDA-MB-330	RPMI	10% FBS, non-essential amino acids and insulin	[26,27]
ZR-75-30	RPMI	10% FBS ^1^, 10 μg/mL insulin	[28]
BT-474	RPMI	10% FBS, Hybri-Care Medium, 1 L cell-culture0grade-water, 1.5 g/L sodium bicarbonate	[102]
MDA-MB-361	RPMI/DMEM	8–10% FBS, 100 U/mL penicillin and 100 μg/mL streptomycin	[101]
UACC-812	L-15/RPMI/DMEM	10–20% FBS, 2 mmol/L glutamine, 1% PSF	[103,104]
MDA-MB-453	RPMI/DMEM	10% FBS, penicillin (100 U/mL), streptomycin (100 μg/mL), 200 mM L-glutamine	[73,105]
HCC1569	RPMI	10% FBS, 2 mM L-glutamine, 100 U/mL penicillin, and 100 μg/mL streptomycin	[75,106]
SUM190PT	Ham’s F12	2% FBS, 1 g/L BSA, 5 mM ethanolamine, 10 mM HEPES, 0.1% hydrocortisone, 5 μg/mL insulin, 50 nM sodium selenite, 5 μg/mL transferrin, 10 nM T3	[107]
AU565	RPMI	10% FBS, 10 mM HEPES, 1 mM sodium pyruvate, 1% penicillin/streptomycin, 2.5 g/L glucose	[107]
SK-BR-3	DMEM/MCCOYS	10% heat-inactivated FBS, 100 μg/mL penicillin G, and 80 μg/mL streptomycin	[108]
SUM229E	RPMI/Ham’s F12	5% FBS, 10 μg/mL, penicillin-streptomycin, 0.5 μg/mL hydrocortisone	[55]
BT-549	RPMI	10% FBS, 100 μg/mL streptomycin, 100 U/mL penicillin, 10 μg/mL insulin	[109]
HCC70	RPMI	10% FBS	[110]
BT-20	EMEM/RPMI/DMEM	10% FBS, penicillin, and streptomycin	[38]
CAL148	DMEM	10% FBS, 1% penicillin-streptomycin, 1% sodium pyruvate	[88]
MDA-MB-157	RPMI/DMEM	10% FBS, 1% 100× penicillin-streptomycin-amphotericin B, 1% 100× nonessential amino acid solution	[7]
MDA-MB-231	RPMI/DMEM	10% FBS, 100 IU/mL penicillin, 100 μg/mL streptomycin	[101]
MDA-MB-435	L-15/RPMI/DMEM	10% heat-inactivated FBS, 100 μg/mL penicillin G, and 80 μg/mL streptomycin	[108]
Hs 578T	RPMI/DMEM	10% FBS, 0.01 mg/mL human insulin	[7,111]

^1^ Foundational media must be used for each breast cancer cell line: EMEM (Eagle’s Minimum Essential Medium), L-15 (Leibovitz’s L-15 Medium), RPMI (Roswell Park Memorial Institute-1640 medium), DMEM (Dulbecco’s Modified Eagle Medium), and Ham’s F12 (Ham’s F-12 (Kaighn’s) Medium). CO2 and air mixture are detrimental to UACC-812 cells when using an L-15 medium for cultivation. Additives are used to ensure the breast cancer cell lines have nutrients to support growth in a controlled environment. Antibiotics are optional for laboratories that would like to prevent bacterial growth. The source for the HCC70 cell line did not add anything to the FBS. FBS: Fetal Bovine Serum, PSF: Penicillin G-streptomycin–fungizone solution, BSA: Bovine Serum Albumin, HEPES (4-(2-hydroxyethyl)-1-piperazineethanesulfonic acid) is a zwitterionic sulfonic acid buffering agent, T3 (Triiodo Thyronine) is a hormone produced by the thyroid gland.

## References

[1] Boekhout A.H., Beijnen J.H., Schellens J.H. (2011). Trastuzumab. Oncologist.

[2] Neve R.M., Chin K., Fridlyand J., Yeh J., Baehner F.L., Fevr T., Clark L., Bayani N., Coppe J.P., Tong F. (2006). A collection of breast cancer cell lines for the study of functionally distinct cancer subtypes. Cancer Cell.

[3] Masters J.R. (2002). HeLa cells 50 years on: The good, the bad and the ugly. Nat. Rev. Cancer.

[4] Holliday D.L., Speirs V. (2011). Choosing the right cell line for breast cancer research. Breast Cancer Res. BCR.

[5] Lasfargues E.Y., Ozzello L. (1958). Cultivation of human breast carcinomas. J. Natl. Cancer Inst..

[6] Burdall S.E., Hanby A.M., Lansdown M.R., Speirs V. (2003). Breast cancer cell lines: Friend or foe?. Breast Cancer Res. BCR.

[7] Cailleau R., Olivé M., Cruciger Q.V. (1978). Long-term human breast carcinoma cell lines of metastatic origin: Preliminary characterization. In Vitro.

[8] Jovanovic J., Rønneberg J.A., Tost J., Kristensen V. (2010). The epigenetics of breast cancer. Mol. Oncol..

[9] Mahgoub M., Monteggia L.M. (2013). Epigenetics and psychiatry. Neurother. J. Am. Soc. Exp. NeuroTher..

[10] Couto J.P., Vulin M., Jehanno C., Coissieux M.M., Hamelin B., Schmidt A., Ivanek R., Sethi A., Bräutigam K., Frei A.L. (2023). Nicotinamide N-methyltransferase sustains a core epigenetic program that promotes metastatic colonization in breast cancer. EMBO J..

[11] Campagna R., Vignini A. (2023). NAD+ Homeostasis and NAD+-Consuming Enzymes: Implications for Vascular Health. Antioxidants.

[12] Sharma M., Tollefsbol T.O. (2022). Combinatorial epigenetic mechanisms of sulforaphane, genistein and sodium butyrate in breast cancer inhibition. Exp. Cell Res..

[13] Mirabelli P., Coppola L., Salvatore M. (2019). Cancer Cell Lines Are Useful Model Systems for Medical Research. Cancers.

[14] Wu H., Ganguly S., Tollefsbol T.O. (2022). Modulating Microbiota as a New Strategy for Breast Cancer Prevention and Treatment. Microorganisms.

[15] Lacroix M., Leclercq G. (2004). Relevance of breast cancer cell lines as models for breast tumours: An update. Breast Cancer Res. Treat..

[16] Dai X., Cheng H., Bai Z., Li J. (2017). Breast Cancer Cell Line Classification and Its Relevance with Breast Tumor Subtyping. J. Cancer.

[17] Hui C., Qi X., Qianyong Z., Xiaoli P., Jundong Z., Mantian M. (2013). Flavonoids, flavonoid subclasses and breast cancer risk: A meta-analysis of epidemiologic studies. PLoS ONE.

[18] Fraga M.F., Ballestar E., Paz M.F., Ropero S., Setien F., Ballestar M.L., Heine-Suñer D., Cigudosa J.C., Urioste M., Benitez J. (2005). Epigenetic differences arise during the lifetime of monozygotic twins. Proc. Natl. Acad. Sci. USA.

[19] Zhou Y., Zheng J., Li Y., Xu D.P., Li S., Chen Y.M., Li H.B. (2016). Natural Polyphenols for Prevention and Treatment of Cancer. Nutrients.

[20] Leroy B., Girard L., Hollestelle A., Minna J.D., Gazdar A.F., Soussi T. (2014). Analysis of TP53 mutation status in human cancer cell lines: A reassessment. Hum. Mutat..

[21] Subik K., Lee J.F., Baxter L., Strzepek T., Costello D., Crowley P., Xing L., Hung M.C., Bonfiglio T., Hicks D.G. (2010). The Expression Patterns of ER, PR, HER2, CK5/6, EGFR, Ki-67 and AR by Immunohistochemical Analysis in Breast Cancer Cell Lines. Breast Cancer Basic Clin. Res..

[22] Ding Z., Xu X., Li T., Wang J., Sun J., Tang L. (2021). ZR-75-1 breast cancer models to study the utility of 18F-FES by PET imaging. Transl. Cancer Res..

[23] Li L.T., Jiang G., Chen Q., Zheng J.N. (2015). Ki67 is a promising molecular target in the diagnosis of cancer (review). Mol. Med. Rep..

[24] Kenny P.A., Lee G.Y., Myers C.A., Neve R.M., Semeiks J.R., Spellman P.T., Lorenz K., Lee E.H., Barcellos-Hoff M.H., Petersen O.W. (2007). The morphologies of breast cancer cell lines in three-dimensional assays correlate with their profiles of gene expression. Mol. Oncol..

[25] Blenkiron C., Goldstein L.D., Thorne N.P., Spiteri I., Chin S.F., Dunning M.J., Barbosa-Morais N.L., Teschendorff A.E., Green A.R., Ellis I.O. (2007). MicroRNA expression profiling of human breast cancer identifies new markers of tumor subtype. Genome Biol..

[26] Tasdemir N., Bossart E.A., Li Z., Zhu L., Sikora M.J., Levine K.M., Jacobsen B.M., Tseng G.C., Davidson N.E., Oesterreich S. (2018). Comprehensive Phenotypic Characterization of Human Invasive Lobular Carcinoma Cell Lines in 2D and 3D Cultures. Cancer Res..

[27] Sflomos G., Schipper K., Koorman T., Fitzpatrick A., Oesterreich S., Lee A.V., Jonkers J., Brunton V.G., Christgen M., Isacke C. (2021). Atlas of Lobular Breast Cancer Models: Challenges and Strategic Directions. Cancers.

[28] Engel L.W., Young N.A., Tralka T.S., Lippman M.E., O’Brien S.J., Joyce M.J. (1978). Establishment and characterization of three new continuous cell lines derived from human breast carcinomas. Cancer Res..

[29] Koseoglu S., Lu Z., Kumar C., Kirschmeier P., Zou J. (2007). AKT1, AKT2 and AKT3-dependent cell survival is cell line-specific and knockdown of all three isoforms selectively induces apoptosis in 20 human tumor cell lines. Cancer Biol. Ther..

[30] Depmap. https://depmap.org/portal/home/#/.

[31] Davey M.G., Hynes S.O., Kerin M.J., Miller N., Lowery A.J. (2021). Ki-67 as a Prognostic Biomarker in Invasive Breast Cancer. Cancers.

[32] Meltzer P., Leibovitz A., Dalton W., Villar H., Kute T., Davis J., Nagle R., Trent J. (1991). Establishment of two new cell lines derived from human breast carcinomas with HER-2/neu amplification. Br. J. Cancer.

[33] Liu X., Ye Y., Zhu L., Xiao X., Zhou B., Gu Y., Si H., Liang H., Liu M., Li J. (2023). Niche stiffness sustains cancer stemness via TAZ and NANOG phase separation. Nat. Commun..

[34] Ruiz-Saenz A., Dreyer C., Campbell M.R., Steri V., Gulizia N., Moasser M.M. (2018). HER2 Amplification in Tumors Activates PI3K/Akt Signaling Independent of HER3. Cancer Res..

[35] Barnabas N., Cohen D. (2013). Phenotypic and Molecular Characterization of MCF10DCIS and SUM Breast Cancer Cell Lines. Int. J. Breast Cancer.

[36] Hollestelle A., Elstrodt F., Timmermans M., Sieuwerts A.M., Klijn J.G., Foekens J.A., den Bakker M.A., Schutte M. (2010). Four human breast cancer cell lines with biallelic inactivating alpha-catenin gene mutations. Breast Cancer Res. Treat..

[37] Castles C.G., Fuqua S.A., Klotz D.M., Hill S.M. (1993). Expression of a constitutively active estrogen receptor variant in the estrogen receptor-negative BT-20 human breast cancer cell line. Cancer Res..

[38] Papadakos K.S., Ekström A., Slipek P., Skourti E., Reid S., Pietras K., Blom A.M. (2022). Sushi domain-containing protein 4 binds to epithelial growth factor receptor and initiates autophagy in an EGFR phosphorylation independent manner. J. Exp. Clin. Cancer Res. CR.

[39] Gu Y., Helenius M., Väänänen K., Bulanova D., Saarela J., Sokolenko A., Martens J., Imyanitov E., Kuznetsov S. (2016). BRCA1-deficient breast cancer cell lines are resistant to MEK inhibitors and show distinct sensitivities to 6-thioguanine. Sci. Rep..

[40] Ziperstein M.J., Guzman A., Kaufman L.J. (2015). Breast Cancer Cell Line Aggregate Morphology Does Not Predict Invasive Capacity. PLoS ONE.

[41] Chiang Y.T., Chien Y.C., Lin Y.H., Wu H.H., Lee D.F., Yu Y.L. (2021). The Function of the Mutant p53-R175H in Cancer. Cancers.

[42] Duraiyan J., Govindarajan R., Kaliyappan K., Palanisamy M. (2012). Applications of immunohistochemistry. J. Pharm. Bioallied Sci..

[43] Riva C., Dainese E., Caprara G., Rocca P.C., Massarelli G., Tot T., Capella C., Eusebi V. (2005). Immunohistochemical study of androgen receptors in breast carcinoma. Evidence of their frequent expression in lobular carcinoma. Virchows Arch. Int. J. Pathol..

[44] Lumachi F., Santeufemia D.A., Basso S.M. (2015). Current medical treatment of estrogen receptor-positive breast cancer. World J. Biol. Chem..

[45] Gajria D., Chandarlapaty S. (2011). HER2-amplified breast cancer: Mechanisms of trastuzumab resistance and novel targeted therapies. Expert Rev. Anticancer Ther..

[46] Li Z., Wei H., Li S., Wu P., Mao X. (2022). The Role of Progesterone Receptors in Breast Cancer. Drug Des. Dev. Ther..

[47] Cellusaurus. https://www.cellosaurus.org/.

[48] Nahleh Z. (2008). Androgen receptor as a target for the treatment of hormone receptor-negative breast cancer: An unchartered territory. Future Oncol..

[49] Niță I., Nițipir C., Toma Ș.A., Limbău A.M., Pîrvu E., Bădărău I.A., Suciu I., Suciu G., Manolescu L.S.C. (2021). Correlation between Androgen Receptor Expression and Immunohistochemistry Type as Prognostic Factors in a Cohort of Breast Cancer Patients: Result from a Single-Center, Cross Sectional Study. Healthcare.

[50] Gucalp A., Traina T.A., Eisner J.R., Parker J.S., Selitsky S.R., Park B.H., Elias A.D., Baskin-Bey E.S., Cardoso F. (2019). Male breast cancer: A disease distinct from female breast cancer. Breast Cancer Res. Treat..

[51] Healey M.A., Hirko K.A., Beck A.H., Collins L.C., Schnitt S.J., Eliassen A.H., Holmes M.D., Tamimi R.M., Hazra A. (2017). Assessment of Ki67 expression for breast cancer subtype classification and prognosis in the Nurses’ Health Study. Breast Cancer Res. Treat..

[52] Scholzen T., Gerdes J. (2000). The Ki-67 protein: From the known and the unknown. J. Cell. Physiol..

[53] Mueller C., Haymond A., Davis J.B., Williams A., Espina V. (2018). Protein biomarkers for subtyping breast cancer and implications for future research. Expert Rev. Proteom..

[54] Hsu J.L., Hung M.C. (2016). The role of HER2, EGFR, and other receptor tyrosine kinases in breast cancer. Cancer Metastasis Rev..

[55] Yu S., Kim T., Yoo K.H., Kang K. (2017). The T47D cell line is an ideal experimental model to elucidate the progesterone-specific effects of a luminal A subtype of breast cancer. Biochem. Biophys. Res. Commun..

[56] Soule H.D., Vazguez J., Long A., Albert S., Brennan M. (1973). A human cell line from a pleural effusion derived from a breast carcinoma. J. Natl. Cancer Inst..

[57] Yu M., Selvaraj S.K., Liang-Chu M.M., Aghajani S., Busse M., Yuan J., Lee G., Peale F., Klijn C., Bourgon R. (2015). A resource for cell line authentication, annotation and quality control. Nature.

[58] Ye L.H., Wu L.Y., Guo W., Ma H.T., Zhang X.D. (2006). Screening of a sub-clone of human breast cancer cells with high metastasis potential. Zhonghua Yi Xue Za Zhi.

[59] Liang Y., Wang S., Liu J. (2019). Overexpression of Tumor Protein p53-regulated Apoptosis-inducing Protein 1 Regulates Proliferation and Apoptosis of Breast Cancer Cells through the PI3K/Akt Pathway. J. Breast Cancer.

[60] Sun X., Kaufman P.D. (2018). Ki-67: More than a proliferation marker. Chromosoma.

[61] Gallardo A., Garcia-Valdecasas B., Murata P., Teran R., Lopez L., Barnadas A., Lerma E. (2018). Inverse relationship between Ki67 and survival in early luminal breast cancer: Confirmation in a multivariate analysis. Breast Cancer Res. Treat..

[62] Badia E., Oliva J., Balaguer P., Cavaillès V. (2007). Tamoxifen resistance and epigenetic modifications in breast cancer cell lines. Curr. Med. Chem..

[63] Zhang L., Wang Q., Zhou Y., Ouyang Q., Dai W., Chen J., Ding P., Li L., Zhang X., Zhang W. (2019). Overexpression of MTA1 inhibits the metastatic ability of ZR-75-30 cells in vitro by promoting MTA2 degradation. Cell Commun. Signal. CCS.

[64] Ma W., Zhu M., Zhang D., Yang L., Yang T., Li X., Zhang Y. (2017). Berberine inhibits the proliferation and migration of breast cancer ZR-75-30 cells by targeting Ephrin-B2. Phytomed. Int. J. Phytother. Phytopharm..

[65] Lasfargues E.Y., Coutinho W.G., Redfield E.S. (1978). Isolation of two human tumor epithelial cell lines from solid breast carcinomas. J. Natl. Cancer Inst..

[66] Akbari A., Akbarzadeh A., Rafiee Tehrani M., Ahangari Cohan R., Chiani M., Mehrabi M.R. (2020). Development and Characterization of Nanoliposomal Hydroxyurea Against BT-474 Breast Cancer Cells. Adv. Pharm. Bull..

[67] Ahmadi-Baloutaki S., Doosti A., Jaafarinia M., Goudarzi H. (2022). Editing of the MALAT1 Gene in MDA-MB-361 Breast Cancer Cell Line using the Novel CRISPR Method. J. Ilam Univ. Med. Sci..

[68] Watrowski R., Castillo-Tong D.C., Obermayr E., Zeillinger R. (2020). Gene Expression of Kallikreins in Breast Cancer Cell Lines. Anticancer Res..

[69] Kilickap S., Kaya Y., Yucel B., Tuncer E., Babacan N.A., Elagoz S. (2014). Higher Ki67 expression is associates with unfavorable prognostic factors and shorter survival in breast cancer. Asian Pac. J. Cancer Prev. APJCP.

[70] Hurst J.H. (2014). Pioneering geneticist Mary-Claire King receives the 2014 Lasker~Koshland Special Achievement Award in Medical Science. J. Clin. Investig..

[71] Darbeheshti F., Izadi P., Emami Razavi A.N., Yekaninejad M.S., Tavakkoly Bazzaz J. (2018). Comparison of BRCA1 Expression between Triple-Negative and Luminal Breast Tumors. Iran. Biomed. J..

[72] Ades F., Zardavas D., Bozovic-Spasojevic I., Pugliano L., Fumagalli D., de Azambuja E., Viale G., Sotiriou C., Piccart M. (2014). Luminal B breast cancer: Molecular characterization, clinical management, and future perspectives. J. Clin. Oncol. Off. J. Am. Soc. Clin. Oncol..

[73] Meng J., Yuan Y., Li Y., Yuan B. (2022). Effects of hirsuteine on MDA-MB-453 breast cancer cell proliferation. Oncol. Lett..

[74] Gazdar A.F., Kurvari V., Virmani A., Gollahon L., Sakaguchi M., Westerfield M., Kodagoda D., Stasny V., Cunningham H.T., Wistuba I.I. (1998). Characterization of paired tumor and non-tumor cell lines established from patients with breast cancer. Int. J. Cancer.

[75] Chung W.P., Huang W.L., Lee C.H., Hsu H.P., Huang W.L., Liu Y.Y., Su W.C. (2022). PI3K inhibitors in trastuzumab-resistant HER2-positive breast cancer cells with PI3K pathway alterations. Am. J. Cancer Res..

[76] Ethier S.P., Duchinsky K., Couch D. (2020). Abstract P4-05-13: The SUM breast cancer cell line knowledge base (SLKBase): A knowledge base and functional genomics platform for breast cancer cell lines. Cancer Res..

[77] Chatterjee N., Wang W.L., Conklin T., Chittur S., Tenniswood M. (2013). Histone deacetylase inhibitors modulate miRNA and mRNA expression, block metaphase, and induce apoptosis in inflammatory breast cancer cells. Cancer Biol. Ther..

[78] Bacus S.S., Kiguchi K., Chin D., King C.R., Huberman E. (1990). Differentiation of cultured human breast cancer cells (AU-565 and MCF-7) associated with loss of cell surface HER-2/neu antigen. Mol. Carcinog..

[79] Bacus S.S., Huberman E., Chin D., Kiguchi K., Simpson S., Lippman M., Lupu R. (1992). A ligand for the erbB-2 oncogene product (gp30) induces differentiation of human breast cancer cells. Cell Growth Differ. Mol. Biol. J. Am. Assoc. Cancer Res..

[80] Di Y., De Silva F., Krol E.S., Alcorn J. (2018). Flaxseed Lignans Enhance the Cytotoxicity of Chemotherapeutic Agents against Breast Cancer Cell Lines MDA-MB-231 and SKBR3. Nutr. Cancer.

[81] Di Modugno F., Mottolese M., DeMonte L., Trono P., Balsamo M., Conidi A., Melucci E., Terrenato I., Belleudi F., Torrisi M.R. (2010). The cooperation between hMena overexpression and HER2 signalling in breast cancer. PLoS ONE.

[82] Jogalekar M.P., Serrano E.E. (2018). Morphometric analysis of a triple negative breast cancer cell line in hydrogel and monolayer culture environments. PeerJ.

[83] Katayose Y., Kim M., Rakkar A.N., Li Z., Cowan K.H., Seth P. (1997). Promoting apoptosis: A novel activity associated with the cyclin-dependent kinase inhibitor p27. Cancer Res..

[84] Zhu M., Liu N., Lin J., Wang J., Lai H., Liu Y. (2022). HDAC7 inhibits cell proliferation via NudCD1/GGH axis in triple-negative breast cancer. Oncol. Lett..

[85] Demeule M., Charfi C., Currie J.C., Larocque A., Zgheib A., Kozelko S., Béliveau R., Marsolais C., Annabi B. (2021). TH1902, a new docetaxel-peptide conjugate for the treatment of sortilin-positive triple-negative breast cancer. Cancer Sci..

[86] Pelicano H., Zhang W., Liu J., Hammoudi N., Dai J., Xu R.H., Pusztai L., Huang P. (2014). Mitochondrial dysfunction in some triple-negative breast cancer cell lines: Role of mTOR pathway and therapeutic potential. Breast Cancer Res. BCR.

[87] Lehmann B.D., Bauer J.A., Chen X., Sanders M.E., Chakravarthy A.B., Shyr Y., Pietenpol J.A. (2011). Identification of human triple-negative breast cancer subtypes and preclinical models for selection of targeted therapies. J. Clin. Investig..

[88] Yamamoto T., Kanaya N., Somlo G., Chen S. (2019). Synergistic anti-cancer activity of CDK4/6 inhibitor palbociclib and dual mTOR kinase inhibitor MLN0128 in pRb-expressing ER-negative breast cancer. Breast Cancer Res. Treat..

[89] Tate C.R., Rhodes L.V., Segar H.C., Driver J.L., Pounder F.N., Burow M.E., Collins-Burow B.M. (2012). Targeting triple-negative breast cancer cells with the histone deacetylase inhibitor panobinostat. Breast Cancer Res. BCR.

[90] Hay R., Park J., Gadzar A. (1994). Atlas of Human Tumor Cell Lines.

[91] Mao L., Wertzler K.J., Maloney S.C., Wang Z., Magnuson N.S., Reeves R. (2009). HMGA1 levels influence mitochondrial function and mitochondrial DNA repair efficiency. Mol. Cell. Biol..

[92] Sheng S., Carey J., Seftor E.A., Dias L., Hendrix M.J., Sager R. (1996). Maspin acts at the cell membrane to inhibit invasion and motility of mammary and prostatic cancer cells. Proc. Natl. Acad. Sci. USA.

[93] Rae J.M., Creighton C.J., Meck J.M., Haddad B.R., Johnson M.D. (2007). MDA-MB-435 cells are derived from M14 melanoma cells--a loss for breast cancer, but a boon for melanoma research. Breast Cancer Res. Treat..

[94] Scherf U., Ross D.T., Waltham M., Smith L.H., Lee J.K., Tanabe L., Kohn K.W., Reinhold W.C., Myers T.G., Andrews D.T. (2000). A gene expression database for the molecular pharmacology of cancer. Nat. Genet..

[95] Robinson T.J., Liu J.C., Vizeacoumar F., Sun T., Maclean N., Egan S.E., Schimmer A.D., Datti A., Zacksenhaus E. (2013). RB1 status in triple negative breast cancer cells dictates response to radiation treatment and selective therapeutic drugs. PLoS ONE.

[96] Li L., Zhang F., Liu Z., Fan Z. (2023). Immunotherapy for Triple-Negative Breast Cancer: Combination Strategies to Improve Outcome. Cancers.

[97] Gianfredi V., Vannini S., Moretti M., Villarini M., Bragazzi N.L., Izzotti A., Nucci D. (2017). Sulforaphane and Epigallocatechin Gallate Restore Estrogen Receptor Expression by Modulating Epigenetic Events in the Breast Cancer Cell Line MDA-MB-231: A Systematic Review and Meta-Analysis. J. Nutrigenet. Nutrigenom..

[98] Ackermann T., Tardito S. (2019). Cell Culture Medium Formulation and Its Implications in Cancer Metabolism. Trends Cancer.

[99] Weiskirchen S., Schröder S.K., Buhl E.M., Weiskirchen R. (2023). A Beginner’s Guide to Cell Culture: Practical Advice for Preventing Needless Problems. Cells.

[100] Soule H.D., Maloney T.M., Wolman S.R., Peterson W.D., Brenz R., McGrath C.M., Russo J., Pauley R.J., Jones R.F., Brooks S.C. (1990). Isolation and characterization of a spontaneously immortalized human breast epithelial cell line, MCF-10. Cancer Res..

[101] Wawruszak A., Luszczki J.J., Grabarska A., Gumbarewicz E., Dmoszynska-Graniczka M., Polberg K., Stepulak A. (2015). Assessment of Interactions between Cisplatin and Two Histone Deacetylase Inhibitors in MCF7, T47D and MDA-MB-231 Human Breast Cancer Cell Lines-An Isobolographic Analysis. PLoS ONE.

[102] Grigoriadis A., Mackay A., Noel E., Wu P.J., Natrajan R., Frankum J., Reis-Filho J.S., Tutt A. (2012). Molecular characterisation of cell line models for triple-negative breast cancers. BMC Genom..

[103] Kao J., Salari K., Bocanegra M., Choi Y.L., Girard L., Gandhi J., Kwei K.A., Hernandez-Boussard T., Wang P., Gazdar A.F. (2009). Molecular profiling of breast cancer cell lines defines relevant tumor models and provides a resource for cancer gene discovery. PLoS ONE.

[104] Finn R.S., Dering J., Conklin D., Kalous O., Cohen D.J., Desai A.J., Ginther C., Atefi M., Chen I., Fowst C. (2009). PD 0332991, a selective cyclin D kinase 4/6 inhibitor, preferentially inhibits proliferation of luminal estrogen receptor-positive human breast cancer cell lines in vitro. Breast Cancer Res. BCR.

[105] Albi E., Mandarano M., Cataldi S., Ceccarini M.R., Fiorani F., Beccari T., Sidoni A., Codini M. (2023). The Effect of Cholesterol in MCF7 Human Breast Cancer Cells. Int. J. Mol. Sci..

[106] Morales Torres C., Wu M.Y., Hobor S., Wainwright E.N., Martin M.J., Patel H., Grey W., Grönroos E., Howell S., Carvalho J. (2020). Selective inhibition of cancer cell self-renewal through a Quisinostat-histone H1.0 axis. Nat. Commun..

[107] Jernström S., Hongisto V., Leivonen S.K., Due E.U., Tadele D.S., Edgren H., Kallioniemi O., Perälä M., Mælandsmo G.M., Sahlberg K.K. (2017). Drug-screening and genomic analyses of HER2-positive breast cancer cell lines reveal predictors for treatment response. Breast Cancer (Dove Med. Press).

[108] Riaz M., van Jaarsveld M.T., Hollestelle A., Prager-van der Smissen W.J., Heine A.A., Boersma A.W., Liu J., Helmijr J., Ozturk B., Smid M. (2013). miRNA expression profiling of 51 human breast cancer cell lines reveals subtype and driver mutation-specific miRNAs. Breast Cancer Res. BCR.

[109] Yamashita N., Morimoto Y., Fushimi A., Ahmad R., Bhattacharya A., Daimon T., Haratake N., Inoue Y., Ishikawa S., Yamamoto M. (2023). MUC1-C Dictates PBRM1-Mediated Chronic Induction of Interferon Signaling, DNA Damage Resistance, and Immunosuppression in Triple-Negative Breast Cancer. Mol. Cancer Res. MCR.

[110] Singha M., Pu L., Stanfield B.A., Uche I.K., Rider P.J.F., Kousoulas K.G., Ramanujam J., Brylinski M. (2022). Artificial intelligence to guide precision anticancer therapy with multitargeted kinase inhibitors. BMC Cancer.

[111] Twomey J.D., Zhang B. (2023). Exploring the Role of Hypoxia-Inducible Carbonic Anhydrase IX (CAIX) in Circulating Tumor Cells (CTCs) of Breast Cancer. Biomedicines.

[112] Puleo J., Polyak K. (2021). The MCF10 Model of Breast Tumor Progression. Cancer Res..

[113] Sabarinathan D., Chandrika S.P., Venkatraman P., Easwaran M., Sureka C.S., Preethi K. (2018). Production of polyhydroxybutyrate (PHB) from Pseudomonas plecoglossicida and its application towards cancer detection. Inf. Med. Unlocked.

[114] DeAngelis J.T., Li Y., Mitchell N., Wilson L., Kim H., Tollefsbol T.O. (2011). 2D difference gel electrophoresis analysis of different time points during the course of neoplastic transformation of human mammary epithelial cells. J. Proteome Res..

[115] Mazzarini M., Falchi M., Bani D., Migliaccio A.R. (2021). Evolution and new frontiers of histology in bio-medical research. Microsc. Res. Tech..

[116] Cailleau R., Young R., Olivé M., Reeves W.J. (1974). Breast tumor cell lines from pleural effusions. J. Natl. Cancer Inst..

[117] Yakavets I., Francois A., Benoit A., Merlin J.L., Bezdetnaya L., Vogin G. (2020). Advanced co-culture 3D breast cancer model for investigation of fibrosis induced by external stimuli: Optimization study. Sci. Rep..

[118] Ryan S.L., Baird A.M., Vaz G., Urquhart A.J., Senge M., Richard D.J., O’Byrne K.J., Davies A.M. (2016). Drug Discovery Approaches Utilizing Three-Dimensional Cell Culture. Assay Drug Dev. Technol..

[119] Katt M.E., Placone A.L., Wong A.D., Xu Z.S., Searson P.C. (2016). In Vitro Tumor Models: Advantages, Disadvantages, Variables, and Selecting the Right Platform. Front. Bioeng. Biotechnol..

[120] Masters J.R. (2000). Human cancer cell lines: Fact and fantasy. Nature reviews. Mol. Cell Biol..

[121] Engel L.W., Young N.A. (1978). Human breast carcinoma cells in continuous culture: A review. Cancer Res..

[122] Mizuno M., Matsuda J., Watanabe K., Shimizu N., Sekiya I. (2023). Effect of disinfectants and manual wiping for processing the cell product changeover in a biosafety cabinet. Regen. Ther..

[123] de Oliveira N.F.P., de Souza B.F., de Castro Coêlho M. (2020). UV Radiation and Its Relation to DNA Methylation in Epidermal Cells: A Review. Epigenomes.

[124] Ben-David U., Siranosian B., Ha G., Tang H., Oren Y., Hinohara K., Strathdee C.A., Dempster J., Lyons N.J., Burns R. (2018). Genetic and transcriptional evolution alters cancer cell line drug response. Nature.

[125] Borg A., Sandberg T., Nilsson K., Johannsson O., Klinker M., Måsbäck A., Westerdahl J., Olsson H., Ingvar C. (2000). High frequency of multiple melanomas and breast and pancreas carcinomas in CDKN2A mutation-positive melanoma families. J. Natl. Cancer Inst..

[126] Janku F., Wheler J.J., Westin S.N., Moulder S.L., Naing A., Tsimberidou A.M., Fu S., Falchook G.S., Hong D.S., Garrido-Laguna I. (2012). PI3K/AKT/mTOR inhibitors in patients with breast and gynecologic malignancies harboring PIK3CA mutations. J. Clin. Oncol..

[127] Cizkova M., Susini A., Vacher S., Cizeron-Clairac G., Andrieu C., Driouch K., Fourme E., Lidereau R., Bièche I. (2012). PIK3CA mutation impact on survival in breast cancer patients and in ERalpha, PR and ERBB2-based subgroups. Breast Cancer Res.

[128] May P., May E. (1999). Twenty years of p53 research: Structural and functional aspects of the p53 protein. Oncogene.

[129] Alkaabi D., Arafat K., Sulaiman S., Al-Azawi A.M., Attoub S. (2023). PD-1 Independent Role of PD-L1 in Triple-Negative Breast Cancer Progression. Int. J. Mol. Sci..

[130] Jiang G., Zhang S., Yazdanparast A., Li M., Pawar A.V., Liu Y., Inavolu S.M., Cheng L. (2016). Comprehensive comparison of molecular portraits between cell lines and tumors in breast cancer. BMC Genom..

[131] Wijshake T., Zou Z., Chen B., Zhong L., Xiao G., Xie Y., Doench J.G., Bennett L., Levine B. (2021). Tumor-suppressor function of Beclin 1 in breast cancer cells requires E-cadherin. Proc. Natl. Acad. Sci. USA.

[132] Haitjema A., Mol B.M., Kooi I.E., Massink M.P., Jørgensen J.A., Rockx D.A., Rooimans M.A., de Winter J.P., Meijers-Heijboer H., Joenje H. (2014). Coregulation of FANCA and BRCA1 in human cells. SpringerPlus.

[133] Légaré S., Chabot C., Basik M. (2017). SPEN, a new player in primary cilia formation and cell migration in breast cancer. Breast Cancer Res. BCR.

[134] Lin J., Ye S., Ke H., Lin L., Wu X., Guo M., Jiao B., Chen C., Zhao L. (2023). Changes in the mammary gland during aging and its links with breast diseases. Acta Biochim. Biophys. Sin..

[135] Kim J.B., O’Hare M.J., Stein R. (2004). Models of breast cancer: Is merging human and animal models the future?. Breast Cancer Res. BCR.

[136] Souto E.P., Dobrolecki L.E., Villanueva H., Sikora A.G., Lewis M.T. (2022). In Vivo Modeling of Human Breast Cancer Using Cell Line and Patient-Derived Xenografts. J. Mammary Gland Biol. Neoplasia.

[137] Okada S., Vaeteewoottacharn K., Kariya R. (2019). Application of Highly Immunocompromised Mice for the Establishment of Patient-Derived Xenograft (PDX) Models. Cells.

[138] Hasan T., Carter B., Denic N., Gai L., Power J., Voisey K., Kao K.R. (2015). Evaluation of cell-line-derived xenograft tumours as controls for immunohistochemical testing for ER and PR. J. Clin. Pathol..

[139] Paul B., Royston K.J., Li Y., Stoll M.L., Skibola C.F., Wilson L.S., Barnes S., Morrow C.D., Tollefsbol T.O. (2017). Impact of genistein on the gut microbiome of humanized mice and its role in breast tumor inhibition. PLoS ONE.

[140] Whittle J.R., Lewis M.T., Lindeman G.J., Visvader J.E. (2015). Patient-derived xenograft models of breast cancer and their predictive power. Breast Cancer Res. BCR.

[141] Han Y., Azuma K., Watanabe S., Semba K., Nakayama J. (2022). Metastatic profiling of HER2-positive breast cancer cell lines in xenograft models. Clin. Exp. Metastasis.

[142] Liu B., Fan Z., Edgerton S.M., Deng X.S., Alimova I.N., Lind S.E., Thor A.D. (2009). Metformin induces unique biological and molecular responses in triple negative breast cancer cells. Cell Cycle.

[143] Georges L.M.C., De Wever O., Galván J.A., Dawson H., Lugli A., Demetter P., Zlobec I. (2019). Cell Line Derived Xenograft Mouse Models Are a Suitable in vivo Model for Studying Tumor Budding in Colorectal Cancer. Front. Med..

[144] Finlay-Schultz J., Jacobsen B.M., Riley D., Paul K.V., Turner S., Ferreira-Gonzalez A., Harrell J.C., Kabos P., Sartorius C.A. (2020). New generation breast cancer cell lines developed from patient-derived xenografts. Breast Cancer Res. BCR.

